# P01 Comparative evaluation of disc diffusion and broth microdilution methods for aztreonam/avibactam susceptibility testing in Enterobacterales

**DOI:** 10.1093/jacamr/dlag102.007

**Published:** 2026-06-26

**Authors:** Ioannis Baltas, Deny Tsakri, Sofia Vourli, Himani Solanki, Evelyn Murrell, Eftychia Kiousi, Zacharias Tsakris, Gabriella Kuczmar, Nikoletta Smyrni, Ioannis Skiadas, Vassilios Grammelis, James Hatcher, Damianos Menegas, Athanasios Tsakris, Georgia Vrioni, Louis Grandjean

**Affiliations:** Infection, Immunity & Inflammation Department, UCL Institute of Child Health, London, UK; Infection, Immunity & Inflammation Department, UCL Institute of Child Health, London, UK; Institute of Biosciences and Applications, NCSR ‘‘Demokritos’’, Athens, Greece; Infection, Immunity & Inflammation Department, UCL Institute of Child Health, London, UK; Infection, Immunity & Inflammation Department, UCL Institute of Child Health, London, UK; Infection, Immunity & Inflammation Department, UCL Institute of Child Health, London, UK; Infection, Immunity & Inflammation Department, UCL Institute of Child Health, London, UK; Department of Clinical Microbiology, Great Ormond Street Hospital for Children, London, UK; Department of Microbiology, Medical School, National and Kapodistrian University of Athens, Athens, Greece; Department of Microbiology, Medical School, National and Kapodistrian University of Athens, Athens, Greece; Department of Microbiology, Medical School, National and Kapodistrian University of Athens, Athens, Greece; Department of Clinical Microbiology, Great Ormond Street Hospital for Children, London, UK; Department of Microbiology, Medical School, National and Kapodistrian University of Athens, Athens, Greece; Pfizer Hellas, Athens, Greece; Pfizer Hellas, Athens, Greece; Infection, Immunity & Inflammation Department, UCL Institute of Child Health, London, UK

## Abstract

**Background:**

Aztreonam/avibactam is a novel β-lactam/β-lactamase inhibitor (BL/BLI) combination active against carbapenem-resistant Enterobacterales (CRE), including MBL-producing isolates. In May 2024, EUCAST published Enterobacterales breakpoints for aztreonam-avibactam. The study aimed to assess the performance of commercially available disc diffusion (DD) against broth microdilution (BMD) using the latest EUCAST breakpoints.

**Methods:**

We tested 278 CRE causing infections in 17 Greek ICUs between 2021 and 2023, using 30/20μg aztreonam-avibactam discs (Mast Group Ltd) and broth microdilution according to EUCAST methodology and EUCAST version 15.0 breakpoints.

**Results:**

Most isolates were identified as *Klebsiella pneumoniae* (97.8%) and 98.9% produced carbapenemases, including 72.7% KPC, 31.3% NDM, 21.6% VIM, 0.7% OXA-48. Using BMD, 94.2% of isolates were susceptible to aztreonam-avibactam. Conversely, using DD, 66.9% were susceptible, 33.1% resistant and 27% within the area of technical uncertainty (ATU). Most isolates in the ATU were KPC-producing (68%) or KPC & MBL-producing (29.3%). All isolates in the ATU (22–24 mm) tested susceptible by BMD (MIC ≤4 mg/L). One isolate exhibited resistance by DD (20 mm) but was susceptible by BMD. Categorical agreement (CA) was 72.7%, with 29% major errors (ME) and 0% very major errors (VME). Using a breakpoint of 22 mm, CA, ME and VME were 99.6%, 0.4% and 0%, respectively.

**Conclusions:**

Aztreonam-avibactam showed potent in vitro activity against MBL- and KPC-producing Enterobacterales. Using the 2025 EUCAST breakpoint, all isolates in the ATU tested susceptible by BMD, leading to high ME of the DD method. A 22 mm breakpoint would have corrected this discrepancy in our cohort.

